# Alcohol and Breast Cancer: The Mechanisms Explained

**DOI:** 10.4021/jocmr2009.07.1246

**Published:** 2009-07-03

**Authors:** Hassen Al-Sader, Hani Abdul-Jabar, Zahra Allawi, Yasser Haba

**Affiliations:** a Department of General Medicine, St Georges Hospital, UK; b Royal National Orthopaedic Hospital Trust, UK; c Queen Mary University of London, UK; d Portsmouth University, UK

## Abstract

Breast cancer is a leading cause of death amongst women, several studies have shown significant association between alcohol consumption and breast cancer. The aim of this overview is to highlight some of the mechanisms by which alcohol consumption could increase the risk of developing breast cancer. Using online Medline search engine, article containing details about mechanisms which explain the link between alcohol and breast cancer were examined. A number of mechanisms were found by which alcohol could increase the risk of breast cancer, alcohol's interaction and effect on oestrogen secretion; number of oestrogen receptors; the generation of acetaldehyde and hydroxyl free radicals; cells migration and metastasis; secretion of IGF1 and interaction with HRT and folate metabolism. In conclusion, it is essential for clinicians to understand these mechanisms and inform patients of the link between alcohol and breast cancer.

**Keywords:**

Breast cancer; Alcohol; Mechanisms

## Introduction

Breast cancer is now the most common cancer in the UK. In 2005 more than 45,500 women were diagnosed with breast cancer, that’s around 125 women a day [1]. Breast cancer is also the leading cause of death amongst women at a 17.4% of total [2]. Risk factors are associated with almost every aspect of women’s lives. Family history, life style, diet, smoking and alcohol are all on the list of increasing the susceptibility to breast cancer.

As the concern for breast cancer is increasing, studies have been conducted in all the different aspects of women’s lives trying to find out what could reduce the risk of breast cancer, and what could increase it. One of those important aspects is alcohol consumption.

A significant association between alcohol intake and breast cancer has been found, with an increase of risk of 7% for each additional 10 grams of alcohol consumed on a daily basis [3].

The aim of this overview is to highlight some of the mechanisms by which alcohol consumption could increase the risk of developing breast cancer.

## Alcohol Metabolism

Ethanol metabolism is often considered to be the predominant factor in the onset of tissue damage, notably through the formation of acetaldehyde. Ethanol metabolism occurs predominantly in the liver, although a variety of other tissues have a significant capacity for ethanol metabolism. Acetaldehyde is the primary product of ethanol oxidation, irrespective of whether it is mediated by aldehyde dehydrogenase (ADH), cytochrome P-450 isoforms, or catalase.

Acetaldehyde is itself oxidised predominantly by means of a highly efficient aldehyde dehydrogenase (ALDH2), localised in the mitochondrial matrix. As a result, both cellular and circulating concentrations of acetaldehyde are maintained in the low micromolar range despite elevated blood ethanol concentrations.

The rate of both ethanol oxidation and acetaldehyde oxidation is determined by the rate of NADH oxidation through mitochondrial electron transport. This activity depends on the supply of other substrates, the demand for ATP in the cell, and the supply of oxygen.

An inadequate electron transport activity would result not only in an inefficient removal of ethanol and acetaldehyde and a highly reduced state of both cytosolic and mitochondrial nicotinamide adenine dinucleotide (NAD), but also promote the overflow of electrons passing through the mitochondrial electron transport chain into the formation of reactive oxygen species (ROS), notably superoxide [4].

## Alcohol and Women

Reporting drinking habits can sometimes be difficult, and so the patient tends not to mention that they drink alcohol regularly or in some cases, the clinician doesn’t even ask.

It is widely reported that women often drink less and have a lower prevalence of drinking problems than men, but this is starting to change. As drinking levels in women begin to approach those in men, rates of drinking problems in women are likely to overtake those of men, as women have greater physiological sensitivity to the effects of alcohol [5].

This physiological sensitivity is probably due to women's lower total body water, gender differences in alcohol metabolism, and effects of alcohol on postmenopausal oestrogen levels. Mortality and breast cancer are increased in women who report drinking more than two drinks daily [6].

Many women have read studies linking hormone replacement therapy (HRT) use with increased breast cancer incidence. It’s due to this that they may avoid HRT. The link between alcohol use and breast cancer risk, in contrast, is at least as strong as that of (HRT), yet little publicity has reached practising physicians or patients [7]. Much of the concentration has been made of smoking, HRT and diet, with alcohol left out.

## Some Evidences for Alcohol Increasing the Risk of Breast Cancer

Two types of research link alcohol and cancer. Epidemiological research has shown a dose-dependent association between alcohol consumption and breast cancer; as alcohol consumption increases, so does risk of developing certain cancers. More tenuous results have come from research into the mechanism by which alcohol could contribute to cancer development. Smith et al [8] found that alcohol intake of at least 30g daily over a period of years increased the risk of breast cancer by 30 to 40% compared with non-drinkers.

Moderate consumption of alcohol (0.07 g/kg) by women who use hormone replacement therapy was found to elevate oestradiol (high levels are risk factors for breast cancer) level beyond exceeding the ovulatory range for four hours [9].

For postmenopausal women even less than one drink a day was associated with up to a 30% increase in breast cancer mortality compared to non-drinkers [10].

In 2004 and their review G. Poschl and H. K. Seitz [11] found that 84% of the 69 case-control and 76% of the 21 cohort studies published so far showed a positive association between ethanol intake and breast cancer.

Analyses included 58 515 women with invasive breast cancer and 95 067 controls from 53 studies. The average consumption of alcohol reported by controls from developed countries was 6.0 g per day, i.e. about half a unit/drink of alcohol per day, and was greater in ever-smokers than never-smokers, (8.4 g per day and 5.0 g per day, respectively). Compared with women who reported drinking no alcohol, the relative risk of breast cancer was 1.32 (1.19-1.45, P < 0.00001) for an intake of 35-44 g per day alcohol, and 1.46 (1.33-1.61, P < 0.00001) for ≥ 45 g per day alcohol.

It is interesting to note that the relationship between smoking and breast cancer was substantially confounded by the effect of alcohol. When analyses were restricted to 22 255 women with breast cancer and 40 832 controls who reported drinking no alcohol, smoking was not associated with breast cancer (compared to never-smokers, relative risk for ever-smokers = 1.03, 95% CI 0.98-1.07, and for current smokers = 0.99, 0.92-1.05). If the observed relationship for alcohol is causal, these results suggest that about 4% of the breast cancers in developed countries are attributable to alcohol [3].

## Proposed Mechanisms for Alcohol Effect

### Alcohol and Oestrogen

Oestrogen is a hormone largely produced (in women) by ovaries. When ovarian production of oestrogen ends, adipose cells are important sites for the conversion of androgenic precursors from the adrenal gland to oestrogen. Moderate alcohol consumption was found to be associated with elevating oestrogen levels in the blood [9].

Oestrogen has many biological effects; amongst them is a proliferative action. Many lines of breast cancer cells depend on oestrogen and progesterone in growth. That means that the breast cells would be exposed to higher levels of oestrogen if women consumed alcohol. This may in turn trigger the cells, which are oestrogen sensitive in these women, to become cancerous.

Alcohol consumption-related increases in oestrogen levels may in turn be partially responsible for the associated decreased risk for coronary artery disease and osteoporosis, as well as for increased risk for breast cancer [12].

Alcohol could increase plasma oestrogen levels either by promoting the induction of aromatases, which can convert androgens to estrogens, or by impairing the metabolism of oestrogen in liver, resulting in oestrogen accumulation in the circulation [13].

This association between alcohol and high oestrogen levels was supported by findings in premenopausal women, as well as postmenopausal [14].

Chronic alcohol intake is associated with ovarian failure, infertility and early menopause, i.e. alcohol has a damaging effect on the female reproductive system. Thus, in premenopausal women, the alcohol effect could be via an effect on the pituitary-ovarian axis [15].

### Alcohol and Oestrogen Receptors

Another suggested mechanism is ethanol effect on level and activity oestrogen receptors on the human breast cancer cells. The oestrogen receptors are members of the steroid hormone receptor family, which are nuclear receptors. They are capable of eliciting a variety of cell responses and influencing signal transduction pathways both in ligand-dependent and ligand-independent manners.

Not all cancer cells have oestrogen receptor. An interesting study was carried by Keith et al [16] to determine whether exposure to ethanol at physiologically relevant concentrations would affect oestrogen receptors of breast cancer cells. The alcohol concentrations used were of moderate to high alcohol intake; i.e. up to three drinks a day and so having blood ethanol level of 5 to 24 mM.

Positive results for cells with oestrogen receptors included: increase in cell proliferation, increased cAMP production, enhanced DNA synthesis, and increased number of oestrogen receptors.

The elevated level of cAMP was suggested as a mechanism for the effects of ethanol on cell proliferation, but the results did not support this, as cAMP levels were not consistent with cell proliferation. Bearing in mind that the ethanol-cAMP relationship is tissue specific and so depends on the tissue examined.

Another proposed mechanism was through affecting oestrogen receptors numbers. Despite the fact that total cell numbers increased at ethanol levels lower than those needed to elevate receptors numbers, the other component for ethanol effect (increasing the receptor activity) is still a possibility.

From this study it might appear that ethanol only affects human cancer cells with oestrogen receptors and not the others. But, there is recent evidence that ethanol intake by women may increase the risk of developing breast tumours with oestrogen or oestrogen and progesterone receptors [17].

### Alcohol and Carcinogenics

Another possibility is that alcohol is converted to carcinogenic substances inside the human body. Oxidation of ethanol to acetaldehyde and the generation of free radicals were studied by Castro et al [18]. Acetaldehyde is a transitional compound in the oxidation of ethanol and acetic acid. It is carcinogenic compound; it is easily oxidised and potentially generating free hydroxyl radicals (carcinogenics). Furthermore, acetaldehyde is also known to be able to inhibit O^6^-methyl guanine demethylase, which is known to be critical for the repair of O^6^-methyl guanine lesions in DNA caused by N-nitrosodimethylamine and of other equivalent alterations resulting from other alkylating carcinogens [10]

The enzymes involved in oxidation of acetaldehyde to acetate are xanthine dehydrogenase (XDh) and xanthine oxidase (XO). Both XOR and AOX can generate reactive oxygen species (ROS), e.g. superoxide anion (O_2_-), hydroxyl radical (OH), and hydrogen peroxide (H_2_O_2_).

ROS cause numerous modifications to DNA, contributing to carcinogenesis and breast cancer.

They need cosubstrates that help them speed the reaction, e.g. NAD+ and caffeine (which is widely available in soft drinks). The study [18] found that incubation of the enzymes with their cosubstrates has significantly increased the generation of acetaldehyde and hydroxyl free radicals.

### Alcohol and Metastasis

In addition for being carcinogenic, and helping in promoting tumour growth via oestrogen levels, alcohol is being investigated for its role in metastasis. Metastasis is the process of tumour cells detaching from primary site and spreading to secondary sites within the human body. It is essential for the cells to detach themselves form the original tissue, be able to attach to extracellular matrix, degradation of extracellular structures and then invade the tissue and the surrounding vessels.

Cells use specific interaction molecules to maintain tissue integrity. Two major groups of these are the E-cadherin (transmembrane glycoprotein) and catenins (cytoplasmic proteins, that link E-cadherin to actin filaments) [19]. In a study conducted by Meng et al [20], in vitro cells were incubated with biologically significant alcohol concentrations (40 - 80 mM). This range of concentrations was calculated at the beginning of the experiment, after no cytotoxicity of alcohol was observed below 100mM.As the first step was detachment of cells from original tissue, treated and untreated cancer cells were lysed in a solution with antibodies for E-cadherin and catenins. Using a detection system, it was found that exposure to alcohol resulted in dose-dependent downregulation of E-cadherin expression. A parallel down expression of catenins was recorded.

For all the other necessary steps for metastasis, it was found that alcohol has a promoting effect of cell adhesion to basement membrane extract and stimulation of cell invasion and migration.

Increasing the motility of cells is essential for cell migration. To examine this, a monolayer of cells was scratched with a sterile tip (a minor wound was created). With alcohol presence, cell flattening and spreading was significantly enhanced along the edges of the wound, compared to untreated samples.

The human breast cancer cells were incubated with Matrigel basement membrane extract. At alcohol concentration of 80mM, adhesion of the cancer cells was significantly enhanced. 95% of cells were attached compared to only 65% in absence of alcohol.

The invasion step is a complex one; it involves secreting enzymes to dissolve the tissue material followed by increased motility. This was tested on invasion of Matrigel basement membrane extract. When 40 and 80 mM of alcohol were present, the invasion increased by approximately 23% and 52% compared with untreated control, respectively.

Even from a protective prospective, alcohol had a negative effect. BRCA1 (breast cancer susceptibility gene 1) is a human gene that is associated with suppression of breast carcinoma [21]. It is only found in low-grade tumours and its absence is associated with high grade (invasive) ones. Alcohol resulted in down expression of BRCA1 gene in a dose dependent manner [20].

### Alcohol and HRT

Another set of studies found that women using the hormone replacement therapy (HRT) and drinking alcohol were at higher risk of developing breast cancer. The extent of impact reached a 3-fold increase in circulating oestradiol on alcohol ingestion (0.7 g/kg/day), for women using HRT [22]. Gapstur [23] has even concluded that the increased risk of breast cancer was confined to this group (rather than users of HRT only).

HRT is the administration of the female hormones oestrogen and progesterone. The use of hormone replacement therapy is highly effective for improving the quality of life of women suffering from acute symptoms of menopause, such as hot flashes, night sweats, insomnia, increased fatigue and irritability. It is also thought that HRT provides some long-term protection against cardiovascular disease and osteoporosis.

The explanation for the study findings is very important. When no explanation could be found, it discourages from emphasising the link between a factor and the studied condition. The same thing happened with alcohol and breast cancer, as no biological explanation could be found for any association, and thus alcohol was not mentioned in some studies that explored HRT and breast cancer. Mills et al [24] described the association as a "nuisance factor" and was viewed as "unexpected and intriguing. Further study is needed...." by Colditz et al [25].

The findings that brought alcohol as the major factor to increase the risk of breast cancer, and not oestrogen supplements (and the fact that there has been no studies that disagreed with those findings), led Zumoff [26] to come up with a hypothesis that states the following: (1) Oestrogen administration to postmenopausal women elevates oestradiol levels modestly. It might increase the level near the thresh hold for breast cancer, but it does not increase the risk unless the patient is genetically susceptible. (2) If alcohol is consumed, women receiving oestrogen replacement therapy will have increased level of oestradiol above thresh hold of breast cancer promoting effects. This is regardless of the genetic background.

### Alcohol and Insulin Like Growth Factors (IGF)

From mammography screening, a peak incidence for duct carcinoma in situ (DCIS) of the breast is seen at the age of 45-50 years followed by a steady decline [27]. It suggests that as the menopause approaches, a high proportion of precancerous lesions in the breast undergo spontaneous resolution. Two mechanisms for that have been proposed: the falling oestrogen level and programmed cell death (apoptosis) [28].

This observation may suggest that mechanisms of breast cancer associated with alcohol are likely to be different in premenopausal from postmenopausal. In postmenopausal disease, it may involve a promoting role of hyperinsulinaemia.

One of the most known consequences of chronic alcohol consumption is liver cirrhosis. Nearly all patients with liver cirrhosis are insulin resistant, β-cells respond to this resistant by increasing insulin levels (hyperinsulinaemia) [29].

In a review by Stoll [30], the elevated levels of the growth hormone/insulin-like growth factor 1 (GH/IGF1) that accompanied hyperinsulinaemia were studied as proposed mechanism for promotion of mammary carcinogenesis.

The liver is the major source of circulating IGF1 and at least six IGF-binding proteins (IGFBPs). The bioavailability of circulating IGF1 is regulated by the different IGFBPs. Whereas IGFBP1 regulates IGF1 availability in response to rapid changes in insulin levels, over 90% of circulating IGF1 is bound to IGFBP3, the latter controls long-term adaptive change to long-term hyperinsulinaemia.

In normal subjects, the effect of insulin is to suppress free IGF1 and IGFBP1 concentrations, but IGFBP3 concentrations are stimulated. But in patients with alcoholic cirrhosis, hyperinsulinaemia decreases IGFBP3 concentrations, resulting in increased IGF1 bioactivity, which stimulates insulin-like growth factor 1 receptor IGF1R activity in mammary tissue cells.

IGF1 has been shown to induce proliferative activity in cells that have undergone transformation [31]. Although these cells could obviously be benign, (IGF1R) is a protein that is thought to have a crucial role in mitogenesis (stimulating mitosis) and transformation to the malignant phenotype.

All the above is through the effect of IGF1, but insulin itself may have a role in mammary carcinoma. Most breast cancer specimens show both IGF1 and insulin receptors. Over-expression of insulin receptors (IR) has been noted in human breast cancer cells and this may make them more responsive to insulin stimulation [32].

Another possible route for IGF1 to affect human breast cancer cells is via its interaction with oestrogen. Both oestrogen and IGF1 are potent mitogens for most human breast cancer cell lines, interaction between their receptors is likely to be involved in modulating the progress of mammary carcinogenesis [26]. In most breast cancer cell lines IGF1 stimulates ER expression, whilst oestrogen increases the expression of IGF1R [33].

The above evidences for IGF1 effects on breast cancer cells, and the how its levels are indirectly affected by alcohol (via hyperinsulinaemia), all that led Stoll [25] to come up with the hypothesis that alcohol only affects postmenopausal women as a promoter for carcinogenesis, via IGF1.

Whilst IGF1 might synergies with oestrogen in a mitogenic effect on mammary epithelium and at the same time prevent apoptosis, IGFBP3 might limit the bioavailability of IGF1 and at the same time increase apoptosis independently of IGF1.

### Alcohol and Folate

The important link between alcohol and folate is that alcohol is a folate antagonist.

This action of alcohol was investigated by Zhang et al [34] in a study to test whether high folate intake (compensating for the alcohol effect) would reduce the risk of breast cancer.

The study found that the risk for breast cancer was higher in women who consumed at least 15g/day of alcohol with low folate intake. High intake of folate had a reducing effect on breast cancer; with at least 600 microgram/day of folate, the risk of breast cancer was reduced by 0.45%, compared with 150-299 microgram/day.

The association between alcohol and breast cancer was observed again; women (with folate intake >300 microgram/day) consuming >15 g/day of alcohol had an 5% increased risk of breast cancer compared with < 15 g/day.

It has long been known that folate can help prevent certain spinal cord birth defects, which is why it is recommended as a supplement for women of child-bearing age.

The protective role of folate from cancer has been studied by Shrubsole et al [35]. The study tested the hypothesis that high intake of folate may reduce the risk of human cancers, including breast cancer.

Folate is a B vitamin found naturally in many foods, including dry beans, peas, and leafy green vegetables, such as spinach. It is essential for regenerating methionine, which is the methyl donor for DNA methylation and for purines and pyramidines required for DNA synthesis. Folate co-factors (vitamin B_6_ and B_12_) have important roles in folate metabolism. Vitamin B_6_ is coenzyme in the conversion of folate to the form used in DNA synthesis, and vitamin B_12_ facilitates methyl transfer.

The body is able to absorb folic acid (used in fortifying foods and in supplements) about twice as easily as the folate found naturally in foods. The recommended daily intake of folate for adults is at least 400 micrograms (ug).

The study evaluated the association between folate and breast cancer in a population who lived in Shanghai, China. It was important that the study is not conducted in a western country. The reason for this is fortification of food. Since 1998, folic acid fortification of cereal food has been mandated in the USA. Therefore studies in North America might lack the appropriate food composition; thus it would not be possible to assess the exact folate intake of the population.

The Shanghai population included women who have diet composed of unfortified and unprocessed food. And they rarely take vitamin supplements that might result in miscalculations of total folate intake. Also in the population, few women drink regularly. This is significant to evaluate the effect of folate, independently from other factors.

The study found that high intake of folate was associated with 0.38 % reduction of breast cancer. Folate co-factors intake was assessed as well. It was found that women with high levels of folate co-factors intake had a 0.53% reduction of breast cancer.

Although the inverse association (between folate intake and breast cancer) was more evident in postmenopausal women, similar pattern of association was found in premenopausal women.

This led Shrubsole et al [35] to come up with several possible mechanisms to explain why folate deficiency might contribute to cancer development: (1) Folate is converted to its active form tetrahydrofolic acid (THF). THF acts as a carrier compound, carrying methyl group (5-mythyl THF) to specific structured that are being synthesised or modified [36]. Methylation is important for gene expression. Folate deficiency may lead to global hypomethylation, which has been linked mutation via genomic instability. (2) Methionine is converted into S-adenosylmethionine (SAM), which is the major methyl group donor in DNA synthesis. When folate level is low, SAM formation may take precedence over thymine synthesis. Low thymine might results in misincorporation of uracil into DNA and higher levels of chromosomal breaks. (3) Although contrary, folate deficiency might result in hypermethylation of specific tumour suppresser genes, reducing their expression.

The alcohol intervention with folate occurs at several aspects of folate transport and metabolism, disrupting its supply to tissues. They include: intestinal absorption, transport to tissue, storage and release by liver [35].

Thus, alcohol could be taken as a factor that reduces folate supply to tissues; this in its turn results in disrupted gene expression and DNA breakage, which are major risk factors for cancer development.

## Conclusions

Breast cancer is the most common cause of cancer death in women world-wide. Epidemiological research has shown a dose-dependent association between alcohol consumption and breast cancer. His overview highlighted the mechanisms by which alcohol consumption could increase the risk of developing breast cancer.

If the observed relationship for alcohol is causal, results suggest that about 4% of the breast cancers in developed countries are attributable to alcohol. Clinicians need to inform women of the link between alcohol and breast cancer, since the use of alcohol is under-reported and rarely explored in depth-even in epidemiological research. The relationship between alcohol and breast cancer should be stressed even for women who do not admit to alcohol use, given the poor rate of intake estimation in most clinical settings.
